# Using the Caries Assessment Spectrum and Treatment Index to Determine the Caries Incidence in Primary and Permanent Molars Among School Children of Kolkata: A Cross-Sectional Study

**DOI:** 10.7759/cureus.35715

**Published:** 2023-03-03

**Authors:** Sujata Kumari, Anupriya Jha, Bhumika Patel, Ankita Sharma, Santhosh Kumar Kuna, Jagdish Rajguru

**Affiliations:** 1 Pediatric Dentistry, Dr R Ahmed Dental College and Hospital, Kolkata, IND; 2 Dentistry, Shyamal Hospital, Patna, IND; 3 Oral Medicine and Radiology, Howard University, Washington, USA; 4 Pedodontics and Preventive Dentistry, Private Practitioner, Bhopal, IND; 5 Oral and Maxillofacial Surgery, St Paul Hospital Millenium Medical College, Addis Ababa, ETH; 6 Oral and Maxillofacial Pathology, Hi-Tech Dental College and Hospital, Bhubaneswar, IND

**Keywords:** index, caries assessment, permanent molars, primary molars, molars

## Abstract

Introduction: Caries in their early stages cannot be properly classified using the decayed, missing, and filled teeth (DMFT) index because it does not record precavitated lesions. Thus, the caries assessment spectrum and treatment (CAST) index is a good alternative as it is equipped to record the whole range of dental illnesses and count restored teeth as healthy ones.

Materials and methods: The participants in this cross-sectional study included 300 children who were seven-to-eight years of age. We used the CAST index to assess the extent of caries in the deciduous and permanent molars of these children. For this, all the permanent and baby teeth, the primary and secondary permanent molars, as well as the first and second deciduous molars were examined to determine the prevalence of each carious stage. The correlation of the distribution of the CAST codes between the first and second molars, the second and first molars, the right and left sides of the dental bend, and the opposing jaws was analyzed using Spearman’s rank correlation coefficient, while the cut-off for statistical significance was a p-value of 0.05. The quantitative analysis was conducted using IBM SPSS Experiences Version 20 for Windows.

Results: By comparing CAST codes in the right and left molars, we were able to observe how the development of caries in paired teeth might affect one another. The rank correlation value was found to be less than 0.5 only in the primary second molars (55/65 and 85/75), which were found exclusively in the deciduous first molars. Moreover, the *r* values for the neighboring deciduous and permanent molars were found to be below 0.3, i.e., 65/64 (0.497), 74/75 (0.327), and 84/85 (0.411), which indicated a weak connection between them. When comparing the teeth in different jaws, we found reasonable correlations (r = 0.33-0.49), with only 64/74 outliers (0.501).

Conclusions: We found that, in the examined population, there was a well-established correlation between the stages of caries development in the deciduous molars on the left side of the mouth.

## Introduction

Dental caries, also known as tooth decay, is a common oral disease globally. It can occur throughout an individual’s lifetime in both deciduous and permanent dentitions. In early childhood, it emerges as the fastest form of tooth decay, affecting the deciduous teeth of infants and toddlers [1]. Dental caries can be prevented by having regular check-ups and avoiding sticky diets, although they are common among children aged 6-19 years [2]. Moreover, the progress of a carious lesion can be managed through a treatment plan that involves a reduction in sugar consumption, the removal of biofilm, the use of fluoride-containing vehicles, and the repair of a cavity in a carious tooth. One alternative treatment is restorative care, which may include pulp therapy. Due to the high cost of these solutions, it is essential to gather data regarding the prevalence of dental caries as well as the treatment requirements to establish the best course of action for preventative care [3].

According to the World Health Organization (WHO), the most widely used caries evaluation criteria only distinguish between the absence and presence of obvious dentine cavities. Thus, the WHO criteria cannot be used to collect information regarding the prevalence of caries in enamel and dentine or whether they affect the pulp or not. Various scientific methods such as indices are used in epidemiologic surveys for evaluating dental caries in both types of dentitions. The decayed, missing, and filled teeth (DMFT) index is one of the easiest-to-use indices; however, it cannot properly classify caries in their early stages because it does not record precavitated lesions [4]. Therefore, the international caries detection and assessment system (ICDAS) was developed as a visual/tactile examination-based caries index that used a two-digit identification system to document restorative and carious status from the earliest detectable change in enamel to the cavity in dentine, although its complex recording standards were a hindrance to its widespread implementation [5]. In 2010, the pulpal involvement, ulceration, fistula, and abscess (PUFA) index was developed to monitor the success of untreated caries. Unlike the DMFT index, the PUFA index may be used to track the progression of untreated caries lesions, which can lead to better outcomes. However, the PUFA index, like the ICDAS, has a weakness in that it focuses on cavitated caries without considering less severe cases [6].

Given the complications and constraints associated with these indices, there is an urgent need for improved indices for the early, treatable stages of caries development. In 2011, Frencken et al. succeeded in this endeavor with the introduction of a cutting-edge caries detection index called the caries assessment spectrum and treatment (CAST) index, which can record the full spectrum of dental illness and count restored teeth as healthy [7]. The excellent specificity, sensitivity, and reproducibility of the CAST index in epidemiological surveys have been confirmed by rigorous in-cell studies validating the assay [8]. The present study set out to determine whether there was a relationship between the stages of caries development in the deciduous and permanent molars among seven-to-eight-year-olds in Kolkata by using the CAST index.

## Materials and methods

The participants in this cross-sectional study were 300 children aged between seven and eight years who were attending public primary schools in Central Kolkata, India. We used the CAST index to assess caries in the deciduous and permanent molars of the participating children over six months between February 2016 and July 2016 after receiving ethical clearance from Dr. R Ahmed Dental College (ethical number IEC/DENT/2016/1). Before carrying out this study, we received permission from the Institutional Ethical Committee along with the approval of the concerned administrators and school principals. The children participating in this study, along with their parents, were provided a clear explanation of the purpose of the study in layman’s terms, after which the parents provided their informed consent.

By using data from comparable Indian studies, a prevalence of caries in deciduous teeth of 70%, a measurement error of 5%, and a 95% confidence interval, we estimated the minimum size of the sample population at 300 (OpenEpi, Version 3), where sample size n = (DEFF*Np [1 - p])/(d2/Z21 - α/2*[N - 1] + p*[1 - p]). Simple random sampling was carried out for the school selection. All the students who were seven-to-eight years of age had four fully erupted permanent molars, could participate in the survey with the consent of their parents, and were present on the day of the survey were included in the study, which made the final sample of 300 children. Conversely, the children with premolars were excluded from the study because the reason for the loss of primary molars (such as exfoliation or missing due to caries) was difficult to determine.

Dental examination

One of us examined the dental checkup according to the CAST guidelines (Table 1). The calculation was performed as suggested by Frenken et al. [7]. Table 1 shows the CAST index describes the full range of carious states in a hierarchical order.

**Table 1:** Description of CAST Codes

Characteristics	Code	Description	Concept of health
Sound	0	There is no outward sign of a carious lesion	Healthy
Sealed	1	Pits and/or fissures are at least partially sealed with a sealant material	
Restored	2	A cavity is restored with a/an (in)direct restorative material	
Enamel	3	Only a noticeable shift in the enamel’s appearance; the discoloration of caries is obvious with or without broken enamel in certain areas	Reversible premorbidity
Dentin	4	Discoloration of the dentin caused by caries on the inside of the tooth; the discolored dentin shows through the enamel, which may or may not have evident regional deterioration	Morbidity
	5	Distinct cavitation into dentin; the pulp chamber is intact	
Pulp	6	The pulp chamber is involved; clear cavitation reaching the pulp chamber or the presence of just root pieces	Serious morbidity
Abscess/ Fistula	7	Involvement of the pulp in a tooth that manifests as a pus-filled bump or a sinus tube that drains pus	
Lost	8	Dental caries caused the tooth to be extracted	Mortality
Other	9	Contradicts the definitions of all other classes	

A dental examination of all teeth was carried out at the school in a room with artificial light, and the teeth were inspected using a dental mirror and a periodontal. For a clean inspection, the children were instructed to properly rinse their mouths with water, and detailed notes were prepared for the condition of each tooth’s outer layer. Having one cavity filled and another enamel lesion on the same tooth was assigned a higher score, while a score of seven was assigned to individuals who had an abscess, fistula, and an open cavity on every surface. The tooth with the highest numerical code was chosen for further examination. About 5% of the analyzed population was re-examined daily to establish intra-examiner dependability.

Statistical analysis

To determine the prevalence of each carious stage, we examined the permanent and baby teeth, the primary and secondary permanent molars, and the first and second deciduous molars. The correlation of the distribution of CAST codes between the first and second molars, the second and first molars, the right and left sides of the dental bend, and the opposing jaws was analyzed using Spearman’s rank correlation coefficient. The cut-off for statistical significance was determined to be p = 0.05. The quantitative analysis was conducted using IBM SPSS Experiences Version 20 for Windows.

## Results

A total of 300 children who were seven-to-eight years of age were examined during the study. Table 2 presents the demographic profile of the study population.

**Table 2:** Demographic profile of the study population

Variables	n (%)
Age	
7 years	100 (33.3)
7.5 years	12 (4.0)
8 years	188 (62.7)
Sex	
Male	114 (38.0)
Female	186 (62.0)
Area	
Rural	178 (59.3)
Urban	122 (40.7)
Total	300 (100)

Out of the 300 total participants, 188 (67.2%), who constituted the majority of subjects, were eight years old, followed by 100 (33.3%) who were seven years old and 12 (4.0%) who were 7.5 years old. Moreover, the participants consisted of 186 (62%) females, which was higher than the number of males, i.e., 114 (38.0%). Besides, the majority of the subjects were from rural areas 178 (59.3%). Table 3 depicts the CAST codes of the evaluated permanent molars.

**Table 3:** Distribution of CAST codes in evaluated permanent molars

CAST INDEX SCORE	16		26		36		46	
	N	%	N	%	N	%	N	%
0	278	92.7	276	92.0	256	85.3	239	79.7
1	0.0	0.0	0.0	0.0	1	0.3	8	2.7
2	0	0.0	0	0.0	0	0.0	0	0.0
3	22	7.3	12	4.0	28	9.3	38	12.7
4	0	0.0	12	4.0	0	0.0	5	1.7
5	0	0.0	0	0.0	11	3.7	8	2.7
6	0.0	0.0	0	0.0	2	0.7	2	0.7
7	0.0	0.0	0	0.0	0	0.0	0	0.0
8	0.0	0.0	0	0.0	2	0.7	0	0.0
9	0.0	0.0	0	0.0	0	0.0	0	0.0

Among the permanent molars, code 3 (i.e., marked change in the enamel only) was the most prominent, while among the four evaluated permanent molars, 46 teeth were found to have the highest percentage of code 3 (12.7%). Code 5 (i.e., distinct cavitation into dentin) was the next most recorded CAST code among permanent molars and was found to have the highest percentage (3.7%) among these 36 teeth. According to different stages of the disease, the distribution of the permanent molars was 88.17% for the teeth in the healthy dentition stage (codes 0-2), 8.33% for the teeth in the reversible pre-morbidity stage (code 3), 9.75% for the teeth in the morbidity stage (codes 4 and 5), 0.33% for the teeth in the serious morbidity stage (codes 6 and 7), and 0.17% for the teeth in the mortality stage (code 8). Table 4 presents the distribution of CAST codes in the evaluated deciduous molars.

**Table 4:** Distribution of CAST codes in evaluated deciduous molars

CAST INDEX SCORE	55		65		75		85		54		64		74		84	
	N	%	N	%	N	%	N	%	N	%	N	%	N	%	N	%
0	204	68.0	203	67.7	186	62.0	185	61.7	221	73.7	213	71.0	204	68.0	200	66.7
1	2	.7	2	.7	7	2.3	0	0	0	0.0	2	0.7	0	0.0	0	0.0
2	0	0.0	0	0.0	0	0.0	0	0.0	0	0.0	0	0.0	0	0.0	0	0.0
3	44	14.7	42	14.0	54	18.0	40	13.3	34	11.3	35	11.7	28	9.3	32	10.7
4	13	4.3	17	5.7	12	4.0	19	6.3	5	1.7	5	1.7	17	5.7	20	6.7
5	18	6.0	21	7.0	14	4.7	24	8.0	10	3.3	17	5.7	24	8.0	21	7.0
6	14	4.7	12	4.0	19	6.3	25	8.3	16	5.3	22	7.3	18	6.0	18	6.0
7	0	0.0	0	0.0	2	.7	0	0.0	2	0.7	0	0.0	2	0.7	0	0.0
8	5	1.7	3	1.0	6	2.0	7	2.3	10	3.3	6	2.0	7	2.3	7	2.3
9	0	0.0	0	0.0	0	0.0	0	0.0	2	0.7	0	0.0	0	0.0	2	0.7

The percentage of the deciduous molars with code 3 (i.e., marked change in the enamel only) was found to be higher than that of permanent molars, and 75 teeth were found to have the highest percentage of code 3 (18%), followed by 55 teeth (14.7%) and 64 teeth (11.7%). Code 6 (i.e., involvement of pulp chamber and the presence of any pathology approaching the pulp chamber or root stumps) was the next most prevalent code, with 85 teeth having the highest percentage (8.3%). According to different stages of the disease, the distribution of primary molars was 67.88% for the teeth in the healthy dentition stage (codes 0-2), 12.88% for the teeth in the reversible pre-morbidity stage (code 3), 10.71% for the teeth in the morbidity stage (codes 4 and 5), 6.25% for the teeth in the serious morbidity stage (codes 6 and 7), and 2.13% for the teeth in the mortality stage (code 8). Table 5 presents Spearman’s correlation matrix along with the p-values.

**Table 5:** Spearman's rho, which measures the degree of similarity between two variables, was calculated for the CAST codes of the teeth that were studied.

Correlations													
		16	26	36	46	55	65	75	85	54	64	74	84
16	p value		.000	.000	.000	.196	.952	.001	.030	.173	.002	.029	.102
26	p value	.000		.000	.000	.553	.206	.097	.926	.147	.406	.969	.265
36	p value	.000	.000		.000	.027	.229	.019	.369	.233	.047	.069	.461
46	p value	.000	.000	.000		.003	.165	.000	.054	.286	.022	.844	.463
55	p value	.196	.553	.027	.003		.000	.000	.000	.000	.000	.000	.000
65	p value	.952	.206	.229	.165	.000		.000	.000	.000	.000	.001	.000
75	p value	.001	.097	.019	.000	.000	.000		.000	.000	.000	.000	.000
85	p value	.030	.926	.369	.054	.000	.000	.000		.000	.000	.000	.000
54	p value	.173	.147	.233	.286	.000	.000	.000	.000		.000	.000	.000
64	p value	.002	.406	.047	.022	.000	.000	.000	.000	.000		.000	.000
74	p value	.029	.969	.069	.844	.000	.001	.000	.000	.000	.000		.000
84	p value	.102	.265	.461	.463	.000	.000	.000	.000	.000	.000	.000	
**. Correlation is significant at 0.01 (two-tailed); *. Correlation is significant at 0.05 (two-tailed)													

**Figure 1:**
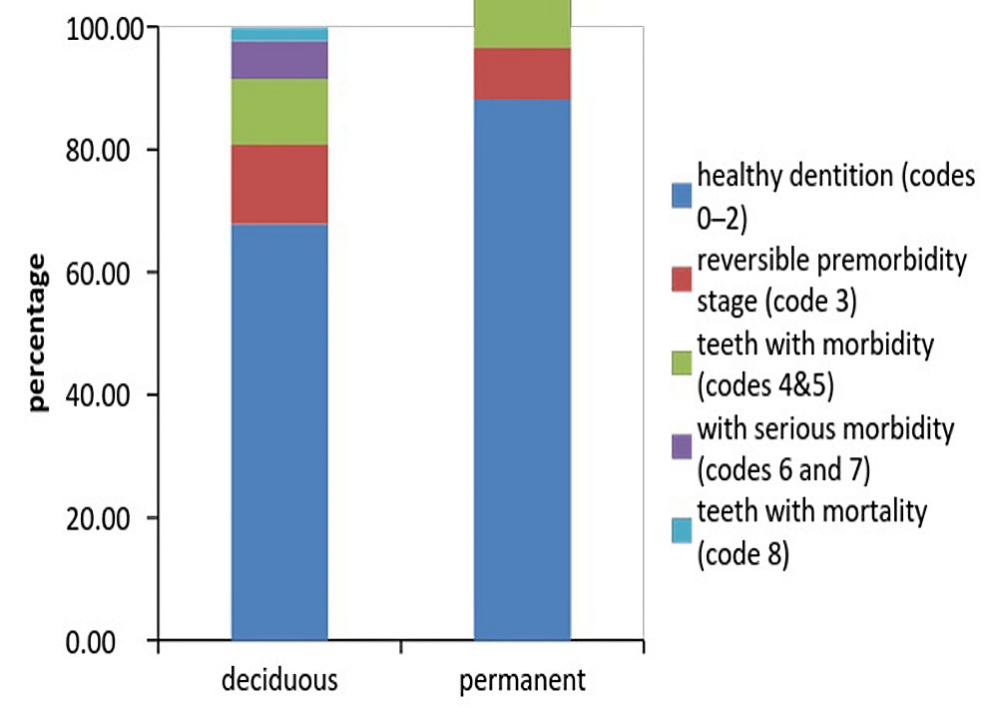
Analyzed molar tooth distribution using the epidemiological health concept

By comparing CAST codes in the right and left molars, we were able to observe how the development of caries in paired teeth might affect one another. The rank correlation value was found to be less than 0.5 only in the primary second molars (55/65 and 85/75), which were found exclusively in deciduous first molars. Further, the r values for the neighboring deciduous and permanent molars were found to be below 0.3, i.e., 65/64 (0.497), 74/75 (0.327), and 84/85 (0.411), indicating a weak connection between them. When comparing the teeth in different jaws, we found reasonable correlations (r = 0.33 to 0.49) with only 64/74 outliers (0.501) (Figure 1).

## Discussion

The purpose of this study was to evaluate the effectiveness of the CAST index, which is a novel caries detection instrument, in identifying caries in deciduous and permanent molars among seven-to-eight-year-old children in Kolkata, India. Since the CAST index offers more in-depth data regarding caries prevalence and experience than the DMFT index does, it is a potential indicator for epidemiological research investigations. The index may be used in a variety of contexts and is designed for widespread implementation, as the validation acceptance ratings for its codes and their explanations have been very high. The index is esthetically pleasing as well as informative, and it has demonstrated excellent levels of agreement between its index codes and the explanations of those codes. Based on the stage of caries development of a tooth and whether it has been filled or not, the CAST index classifies the tooth as either sound or decayed. Using this index is superior to using the ICDAS II, PUFA, and DMF indices separately because the CAST index allows for the coverage of the whole range of carious lesion development, facilitating the dental management of an entire population [7].

Whereas there are three categories for lacquer caries sores in the ICDAS framework, there is only one in the CAST index. Therefore, when assessing the current state of caries according to the CAST index, it is helpful to introduce a pre-bleakness stage for future preventative measures. Moreover, a project log can help in grading early-stage dentin sores when they are most amenable to treatment [5]. However, the inactive and active lesions are sometimes not distinguished in the CAST index, and neither the validity nor the trustworthiness of the CAST index has been established. Further, it lacks the data necessary for clinical studies such as information regarding treatments and preventative actions that may be applied to each code. Furthermore, whereas three stages of enamel damage are found in the ICDAS framework, there is only one in the CAST index. Therefore, to aid in preventative treatment, caries activity can be described using the CAST index, after which a pre-morbidity stage can be assigned to it.

The three indicators were combined to form the new CAST index, which encompasses the whole range of dental caries, from the absence of any carious lesion to the most advanced phases of caries lesion development in the pulp and surrounding tissue, including caries protection (sealant) and caries cure (restoration). Restored teeth are placed at the top of the list because, unlike typical caries indices, they are deemed to be healthy and fully functional. The CAST index is useful for assessing the potential success of early-stage treatment for dentin lesions [9].

In our study, we observed the involvement of the pulp chamber. In 8.3% of evaluated deciduous teeth, this stage was deemed the most dangerous because of the existence of obvious cavitations extending toward the mash chamber or the presence of only root fragments. By the time a child is seven or eight years old, the components that cause dental caries have acted too briefly to trigger the development of severe cavities in permanent teeth. Due to their lower thickness and a considerably larger mash chamber, essential teeth are prone to a faster and more painful transfer from finish to dentine and then to the development of pulpitis than exceptionally sturdy teeth.

Globally, the dental treatment of deciduous dentition has been neglected. According to several previous studies, the PUFA index, which measures the progression of untreated dental caries, has been proven to have a favorable correlation with the DMFT level [10]. As the front teeth are constantly being switched around among seven-to-eight-year-olds, this study focused on the relationships between molar statuses. We were able to maintain a more consistent sample size by excluding incisors and canines from the statistical analysis.

We found the prevalence of caries to be higher in deciduous molars than in permanent molars, which is consistent with the findings of previous studies focusing on other populations [11-13]. Moreover, we found the lowest prevalence of code 3 for the first essential molars, even though the prevalence of code 3 between the second deciduous and the first permanent molars was found to be comparable. Further, we observed that cavitated sores were more common in the important molars than in the super-durable molars. This is in line with the study by Honcalaet et al. on Estonian children, which found that 17% of first permanent molars had enamel lesions present on the occlusal surface of wet teeth (ICDAS code 2) [14]. This may be because the variables producing dental caries have too little of an effect at the age of seven or age years to alter the formation of deep cavities in permanent teeth. When compared to permanent teeth, primary teeth have a quicker rate of lesion development from enamel to dentine, which leads to pulpitis. Neglect in the care of primary teeth is another possible cause of this, and this is a problem in many parts of the world [15-19].

In our study, the first molars exhibited the greatest rates of tooth death and significant morbidity (codes 5 and 6, i.e., involvement of pulp and tooth surrounding tissues). This finding contradicts several other studies that found the second primary molars to be more impacted than the first primary molars. Lesions from caries are more likely to form on the occlusal (chewing) surface of the permanent molars and the buccal (chewing) pit of the lower molars. Therefore, high-risk groups may consider getting sealants [20,21]. In both the main and secondary sets of teeth, we used the CAST index to determine that the contralateral molars were related [22,23]. Our analysis suggests that the hypothesized aggregate caries design holds if one considers the stronger links between the CAST classes observed in the adjacent important molars on the left side of the mouth [24].

The limitations of the study were that the CAST index does not account for the difference between the active and passive phases of carious lesions in enamel. Although any enamel carious lesion should be included in an index, it should not be included in caries epidemiological surveys.

## Conclusions

The CAST index has introduced a new paradigm shift from curative to preventive dentistry. In the examined population, we found a well-established correlation between the stages of caries development in deciduous molars on the left side of the mouth. This study highlights the benefits of the CAST index in epidemiological investigations. However, a clinical preliminary should not use the CAST index to track the progression of carious lesions.
